# LOH Detected by Microsatellite Markers Reveals the Clonal Origin of Recurrent Laryngeal Squamous Cell Carcinoma

**DOI:** 10.1371/journal.pone.0111857

**Published:** 2014-11-03

**Authors:** Zhaoyang Cui, Xinliang Pan, Qirong Wang

**Affiliations:** 1 Department of Otorhinolaryngology, Shandong Provincial Qianfoshan Hospital, Clinical Medical College of Shandong University, Jinan, China; 2 Department of Otorhinolaryngology, Qilu Hospital, Shandong University, Jinan, China; University of North Carolina School of Medicine, United States of America

## Abstract

**Background:**

The question of whether “recurrent” laryngeal carcinoma is truly a new tumour with a clonal origin that differs from that of the primary tumour has remained unanswered. The objective of this study was to determine whether recurrent tumours have the same genetic basis as primary tumours, as the answer to this question is important for the development of treatment strategies.

**Materials and Methods:**

Matched samples consisting of primary tumour, recurrent tumour and normal tissue were obtained from the same patient. A total of 37 patients with laryngeal cancer were examined for loss of heterozygosity (LOH) on the 3p, 5p, 7q, 8p, 9p, 13p, 17p and 18q chromosomal arms using PCR to amplify microsatellite markers. All patients were routinely followed up and 5-year survival rates were calculated using directly calculating method and Kaplan-Meier's method.

**Results:**

A total of 28 out of 37 (75.6%) patients showed LOH at a minimum of one locus, and 19 out of 37 (51.3%) patients showed LOH at two loci. Primary and recurrent tumours in each patient showed identical allelic loss patterns and incidence rates. Patients without LOH had a longer average time to recurrence than patients with LOH (P<0.05). Additionally, patients with LOH had a longer average smoking duration prior to surgery than patients without LOH (P<0.05). The 5-year survival rates were 32.14%in patients with LOH versus 44.4% in patients without LOH.

**Conclusions:**

The data indicate that primary and recurrent tumours have the same clonal origin. This result implies that we failed to radically resect the primary tumours and/or micrometastases in these patients. Consequently, some form of adjunctive therapy may be necessary. Additionally, the data indicate that the recurrence of laryngeal squamous cell carcinoma is closely related to chromosomal aberrations (specifically LOH).

## Introduction

Laryngeal carcinoma is a common malignant tumour of the head and neck in many countries, including northern China [1], [2]. Despite continuous improvements in various surgical procedures and the introduction of new chemotherapies with mild side effects, morbidity and mortality have increased significantly in patients with laryngeal carcinoma [3]. Many of these patients showed local recurrence after surgery, regional spread and even distant metastases [4]. Postoperative laryngeal carcinoma recurrence is the major cause of treatment failure [5]. The recurrence site can be local or distant; alternatively, it can be located in regional lymph nodes [6],[7]. There are two primary reasons for recurrence: the first is that the primary tumour or the micrometastasis was not completely resected, which allows rapid tumour recurrence at the site, while the second is that the recurrent laryngeal carcinoma is actually a new tumour with a clonal origin that differs from that of the primary tumour. It is important to determine whether the “recurrent” laryngeal carcinoma is a true relapse or a new tumour. This distinction has important clinical significance not only for the study of head and neck tumourigenesis but also for the determination of a therapeutic strategy. The treatment of patients with recurrent tumours is an important problem that physicians face. Thus, further understanding of the association between biological features and the prognosis of recurrent laryngeal carcinoma and primary tumours may provide a better theoretical foundation for the rational and standardised treatment of laryngeal carcinoma.

Loss of heterozygosity (LOH) is the most common phenomenon in the inactivation of putative tumour suppressor genes, and LOH analyses have provided important clues that have helped to confirm the position of putative tumour suppressor genes and to elucidate the cloning process [8]. Microsatellite DNA can be stably inherited from one generation to the next. LOH analysis may be helpful in differentiating synchronous primary ovarian and endometrial cancer from a single tumour that has metastasised [9]. Ng et al. [10] compared the utility of microsatellite LOH, P53 mutation types and integration modes of HBV-DNA to determine the clonal relationships of multiple tumour nodules in hepatocellular carcinoma (HCC) patients and concluded that LOH analysis can be used to evaluate tumour clonality. Zhu et al. used LOH analysis to determine that two closely neighbouring HCCs appeared to be intrahepatic metastases of double primary tumours [11]. LOH also has numerous implications for human genetic identification [12]. Frequent allelic loss at chromosomal locations 2q, 3p, 4q, 6p, 6q, 8p, 8q, 9p, 11q, 13q, 14q and 17q is observed in head and neck cancer patients [13]–[16]. Sharma et al. reported a patient with squamous cell carcinoma of the larynx and mandible who underwent LOH testing to clarify the clonal relationship. These authors concluded that testing for LOH can be useful for differentiating distant metastases from a second primary cancer, thereby providing important prognostic and staging information [17]. Lung tumours and head and neck tumours that develop in a localised region also tend to have a common clonal origin; [17] therefore, LOH testing can also be useful for human genetic identification or to determine the clonal origin of a recurrent tumour. Our study attempts to answer the question of whether “recurrent” laryngeal carcinoma is a true recurrence or a new tumour with a clonal origin that differs from that of the original primary tumour. Because chromosomal deletions can affect more than one gene, in this study, we selected 10 different microsatellite markers based on their high incidence as reported in the literature, and we determined whether mutations in primary tumours match with those identified in recurrent tumours in the same patient.

## Materials and Methods

The study population consisted of 62 male patients who underwent surgical resection of primary laryngeal carcinoma and “recurrent” laryngeal carcinoma at Qilu Hospital of Shandong University or Shandong Provincial Qianfoshan Hospital between January 2005 and January 2010. All clinical and pathological data (i.e., age, marital status, allergies, smoking and drinking histories, laryngoscopy results, biopsy specimens, magnetic resonance imaging [MRI] and paraffin-embedded tissue) were obtained. Twenty-five of the 62 registered patients were excluded because of a lack of tissue specimens or poor quality of the DNA obtained from the specimens. In the remaining 37 patients, we identified the following tumours based on the World Health Organization histological classification of tumours: 29 well-differentiated (w/d), 7 moderately differentiated (m/d) and 1 poorly differentiated (p/d). All patients were routinely followed up and 5-year survival rates were calculated using directly calculating method and Kaplan-Meier's method.The deadline is June,2014.The tumour sample collection and data collection protocols were approved by the Ethics Committees of Qilu Hospital of Shandong University and Shandong Provincial Qianfoshan Hospital, and all patients provided signed informed consent.

### Laser capture microdissection, DNA extraction, and DNA amplification

Laser capture microdissection (LCM) is an innovative technique that allows the isolation and enrichment of pure subpopulations of cells from tissue under direct microscopic examination. The material obtained by LCM can be used for downstream assays, including microarray, Western blotting, cDNA library generation and DNA genotyping. Thus, we used LCM for cell collection following a previously described protocol [18], [19]. Paraffin-embedded sections (10 µm thickness) were stained with Gill's haematoxylin, which limits DNA damage.

For the paraffin-embedded specimens, laryngeal carcinoma, recurrent laryngeal carcinoma and normal tissue were obtained from the same patient. Tumour histology was confirmed by a pathologist. Paraffin-embedded sections were stained with Gill's haematoxylin and subsequently deparaffinised and microdissected. DNA was extracted according to the instructions provided with the DNA Extract Kit (OMEGA, USA), and the sample was considered to be of sufficient quality and quantity when the OD260/OD280 ratio was between 1.8 and 2.0 and the DNA concentration was higher than 50 ng/µl. The primers designed to amplify each microsatellite marker are shown in Table 1. Matched DNA samples obtained from primary tumour, recurrent tumour, and normal tissues were amplified with the respective primer pairs under standard PCR conditions. Taq DNA polymerase can introduce sequence errors during PCR amplification, and unequal amplification of the two alleles can result in false-positive detection of LOH. Therefore, both the normal and tumour DNA templates were amplified in three independent PCR reactions. Three PCR products per reaction were analysed on a 1% agarose gel by electrophoresis, and we purified the PCR product with the brightest band. Because of the possibility that the tumour tissue was contaminated with normal stroma, allelic loss in informative patients was defined as a >50% decrease in the ratio of polymorphic band intensities in the tumour tissue versus that in the surrounding normal stromal tissue.

**Table 1:** Microsatellite markers selected for detection.

Locus	Forward Primer and Reverse Primer
D8s261	Forward Primer: TGCCACTGTCTTGAAAATCC
	Reverse Primer: TATGGCCCAGCAATGTGTAT
D9s171	Forward Primer: GCTAAGTGAACCTCATCTCTGTCT
	Reverse Primer: ACCCTAGCACTGATGGTATAGTCT
D9s104	Forward Primer: ACTGGGACTCTAACTAATGT
	Reverse Primer: GATCTGGGTATGTCTTTCTG
D13S317	Forward Primer: ACAGAAGTCTGGGATGTGGA
	Reverse Primer: GCCCAAAAAGACAGACAGAA
D8S552	Forward Primer: AGGATTGTAATTTCCTTGC
	Reverse Primer: GGGACTTTTTGAAGGTTTG
D9S162	Forward Primer: GCAATGACCAGTTAAGGTTC
	Reverse Primer: AATTCCCACAACAAATCTCC
TP53	Forward Primer: GTGTTATCTCCTAGGTTGGC
	Reverse Primer: AGACTTAGTACCTGAAGGGT
D3S1300	Forward Primer: AGCTCACATTCTAGTCAGCCT
	Reverse Primer: GCCAATTCCCCAGATG
D3S1234	Forward Primer: CCTGTGAGACAAAGCAAGAC
	Reverse Primer: GACATTAGGCACAGGGCTAA
D5S592	Forward Primer: AGACAGACAGAGAGATTAGA
	Reverse Primer: AGTAAAGTGAGTGGAGAGC

Three microliters of purified DNA was used in each PCR reaction in a final volume of 30 µl. In addition to the DNA template, the mix consisted of 15 µl of EasyTaq PCR SuperMix (TransGen Biotech), 0.4 µl of each of the forward and reverse primers, and ddH_2_O up to a final volume of 30 µl. The PCR conditions were as follows: an initial denaturation at 95°C for 10 min, followed by 30 cycles of amplification with denaturation at 95°C for 90 s, primer annealing for 90 s at a temperature specific for each marker (i.e., in the range of 44–59°C), and elongation at 72°C for 90 s. Then, the amplified PCR products were electrophoresed on a 1% agarose gel, after which the products were separated on an 8% polyacrylamide urea gel to detect the microsatellite markers. The gels were then silver stained. When LOH was observed, the experiment was repeated again.

The results were visually interpreted by two independent observers, and any discrepancies were resolved by discussion. LOH was determined by the decrease in intensity of one allele relative to the other, and intensities were compared in the tumour and in the normal tissues by visual inspection. Patients who were homozygous throughout the length of all of the microsatellite alleles were considered to be uninformative.

### Statistical analyses

All patients were followed in letters and telephone calls. Survival time was calculated from the date of first visit to our hospital to the date of death from Laryngeal carcinoma. Surviving patients were censored as of the last date on which they were known to be still alive. Survival estimates were calculated by using the Kaplan-Meier method.The data were analysed using the Statistical Package for Social Sciences (SPSS for Windows, version 17.0; SPSS, Chicago, IL, USA). Between-group differences in baseline characteristics were determined using the t-test, and the data are presented as the mean±standard deviation (mean±SD).Probability values of less than 0.05 were considered statistically significant.

## Results

In this study, we performed LOH analysis using 37 patients with laryngeal squamous cell carcinoma. A total of 75.7% (28/37) of these patients had detectable LOH. LOH was found at two loci in 19 patients and one locus in 9 patients; the remaining patients did not show detectable LOH. The LOH frequencies at D8s261, D9s171, D9s104, D13S317, D8S552, D9S162, P53, D3S1300, D3S1234 and D5S592 were 24.3%, 62.2%, 56.7%, 29.7%, 21.6%, 37.8%, 32.4%, 40.5%, 48.6% and 45.9%, respectively. A greater frequency of LOH was found for D9s171 (62.2%), D9s104 (56.7%), D3S1234 (48.6%), D5S592 (45.9%) and D3S1300 (40.5%) compared to the other markers. The LOH frequency data for each microsatellite marker are summarised in Table 2.

**Table 2:** Percentages of LOH at each microsatellite marker.

Microsatellite marker	LOH in primary tumour	LOH in recurrent tumour
D3S1234	48.6%(18/37)	48. 6%(18/37)
D5S592	45.9%(17/37)	45.9%(17/37)
D9S171	62.2%(23/37)	62.2%(23/37)
D9s104	56.7%(21/37)	56.7%(21/37)
D9S162	37.8%(14/37)	37.8%(14/37)
D3S1300	40.5%(15/37)	40.5%(15/37)
D8s261	24.3%(9/37)	24.3%(9/37)
D8S552	21.6%(8/37)	21.6%(8/37)
TP53	32.4%(12/37)	32.4%(12/37)
D13S317	29.7%(11/37)	29.7%(11/37)

The data show that the average time to recurrence was 13.17±5.27 months in the LOH group, while the average time to recurrence was 35.56±11.10 months in the non-LOH group. This difference is statistically significant (P<0.05), Table 3.

**Table 3:** The relationship among LOH, the average duration of smoking, and the average time to recurrence.

Number of cases	Average time to recurrence (months)	P	Average duration of preoperative smoking (months)	P
LOH (28)	13.17±5.27	0.0032	233.57±92.02	0.0005
No LOH (9)	35.56±11.10	P<0.01	107.56±63.19	P<0.01

The average duration of preoperative smoking was 107.56±63.19 months in the non-LOH group and 233.57±92.02 months in the LOH group. This difference is statistically significant (Table 3).

The baseline characteristics of the LOH and non-LOH groups were compared using the chi-square test. Note that the two groups had similar baseline characteristics (Table 4).

**Table 4:** Comparison of the baseline characteristics between the groups.

Characteristics	LOH group (28)	No LOH group (9)	P
Age (median)	65.3	66.7	>0.05
Male gender	28	9	>0.05
Smoking habit	28	9	>0.05
Drinking habit	23	6	>0.05
Tumour location			>0.05
Larynx	21	8	
Hypopharynx	7	1	
Lymph node Metastasis	16	5	>0.05
TNM II	2	1	>0.05
III	26	8	

**Figure 1:**
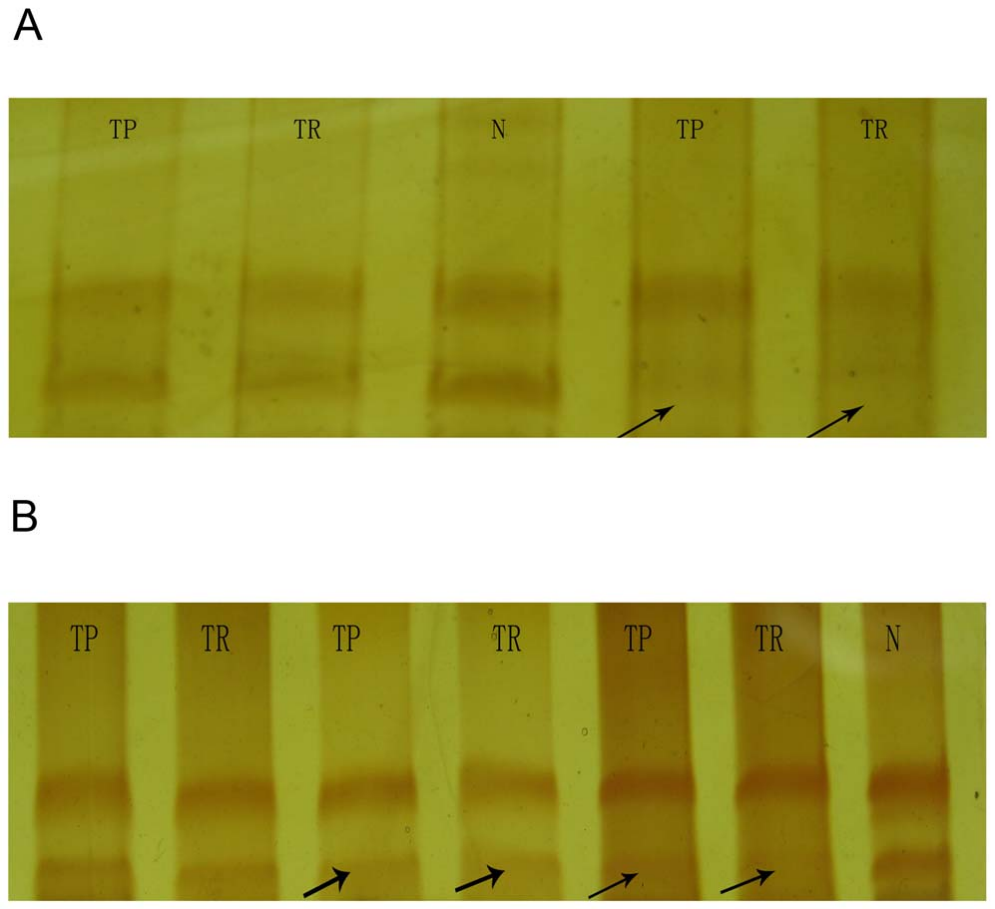
An LOH-mode electrophoresis diagram. A) A representative laryngeal LOH-mode electrophoresis diagram at D5S592. B) A representative laryngeal LOH-mode electrophoresis diagram at D9S171. TP, primary tumour; TR, recurrent tumour; N, normal tissue. The arrow indicates the missing band. The normal tissue was derived from areas adjacent to the tumour and served as a control.

The pairing of cancer tissues and normal tissues shows that these two tissue types have the same areas of microsatellite homozygosity (Figure 1), which may be why some researchers have predicted that primary tumours and recurrent tumours have the same clonal origin in the same patient.

**Figure 2:**
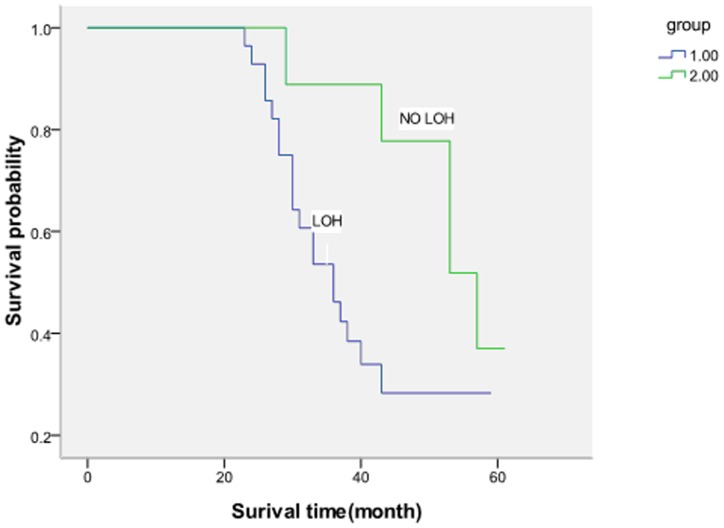
Overall survival of patients without LOH and patients with LOH (P = 0.021).

According to the study, 13 out of 37 patients were still alive. One patient had been followed up for 120 months, unfortunately, we found that tumor recurred last week.10 patients died from respiratory difficulty, 7 patients died from cachexia, 2 patients died from hemorrhage, In 5 patients the cause of death is not clear. Using directly calculating method and Kaplan-Meier's method, the 5-year survival rates were 32.14%(9/28) in patients with LOH versus 44.4%(5/9) in patients without LOH. A significant difference between patients with LOH and patients without LOH(p = 0.021) (Figure 2). The prognosis was best of patients without LOH and worst of patients with LOH.

## Discussion

The prevalence of metastases has continued to increase in patients with head and neck squamous cell carcinoma [17]. A pertinent question is whether “recurrent” laryngeal carcinoma is a true relapse or a new tumour. Clinicopathologic evidence has been the gold standard for distinguishing distant metastases from second primary tumours [17]; however, this method is relatively subjective. Yoo suggested that chromosomal alterations may be involved in the carcinogenesis of laryngeal carcinomas [16]. LOH in cancer can occur either because of chromosomal deletions or somatic recombinations that result in uniparental disomy [20]. Dos [21] implicated different tumour suppressor genes that map to distinct regions in oral and laryngeal carcinomas. Shiga [22] observed that the functions of tumour suppressor genes differ among HNSCC patients living in various regions and that allelic loss plays a key role in the acquisition of a malignant phenotype in these tumours. Shiga [23] also observed that field carcinogenesis caused by the exposure of oral, laryngeal and pharyngeal mucosa to carcinogens may lead to the independent development of synchronous, non-related tumours. Because chromosomal deletions can affect more than one gene, we included 10 different microsatellite markers in our study. The high frequency of LOH that was observed in the head and neck tumours in this study suggests that the phenomenon of “field cancerization” [24] first described by Slaughter in 1953 may partially result from the clonal proliferation of mucosal epithelial cells with allelic loss. Our data support this hypothesis.

Our study provides evidence that primary tumours and recurrent tumours in the same patient have identical patterns of allelic loss. This evidence implies that primary and recurrent tumours have the same clonal origin, and it also implies that we failed to radically resect primary tumours and/or micrometastases in these patients. Indeed, complete resection of a micrometastasis is difficult because we are unable to see micrometastases with the naked eye. Hamakawa [25] detected SCCA tumour mRNA by nested PCR in 37 of 198 histologically metastasis-negative nodes. Yoshioka [26] established a sensitive and rapid genetic assay to detect cancer micrometastases and observed that 10 of 50 patients were scored as node-positive by haematoxylin and eosin (H&E) stain, whereas 24 patients were scored as node-positive by genetic diagnosis. A histological and molecular analysis was performed using a single MPNST tumour that was subdivided into three histopathologically distinct regions: a benign PNF (region 1), an atypical PNF (region 2) and a high-grade MPNST (region 3). The NF1-associated LOH analysis revealed that LOH was increased in the three tumour types, with 9%, 42% and 97% LOH evident in regions 1, 2 and 3, respectively [27]. Zhao [28] examined primary tumours and 182 neck lymph nodes from twenty patients using dissection of supraglottic cancer with immunohistochemical staining (i.e., anti-cytokeratin 19 and H&E staining). The authors observed that CK19 expression was present in 23.6% of the lymph nodes and all of the primary tumours. The H&E staining results showed that 16.5% of the lymph nodes were positive for tumour cells, which represented a highly significant difference.

LOH in surgical margins may predict local relapse in head and neck squamous cell carcinoma [29], as it does in gastrointestinal stromal tumours [30]. Meanwhile, the presence of LOH in the primary tumour may also be predictive of lymph node metastasis [31] and poor prognosis [32]. Previous studies have revealed a significant association between LOH and cancer stage [33]; however, the study by Szukala et al. did not reveal the predictive value of LOH with respect to local relapse in patients with laryngeal carcinoma [25]. We demonstrate herein that LOH in patients with laryngeal carcinoma is associated with a poor prognosis. According to the results, we divided the patients into an LOH group and a non-LOH group and compared the average time to recurrence in the two groups. We found that the non-LOH group had a longer average time to recurrence than the LOH group. This difference was statistically significant, which indicates that the recurrence of laryngeal carcinoma is closely related to chromosomal aberrations (LOH). Genomic aberrations were observed more often in the patients with greater histologic grades and higher disease stages [34]. We believe that the association between recurrence of laryngeal carcinomas and the occurrence of LOH can be used as a reference index to predict postoperative recurrence.

Our findings imply that patients with laryngeal carcinoma may need adjunctive therapy if their primary tumours or micrometastases are not completely resected. Combined treatment for advanced head and neck cancer is more efficacious and is no more toxic than hyperfractionated irradiation alone. Clinical trials have shown improvements in the relapse-free survival, locoregional control and overall survival rate for patients randomised to combined modality therapy [35]. These findings are consistent with other published results. Jamieson [36] observed that patients with an intact M6P/IGF2R had similar prognoses regardless of whether they received radiotherapy alone or combined modality treatment. Conversely, head and neck cancer patients with M6P/IGF2R LOH would benefit most from a combined treatment modality. This scenario would be desirable because the morbidity of patients who receive combined modality therapy is greater than that of patients who receive radiotherapy alone [37], [38]. This result implies that LOH may help to identify a group of patients with laryngeal carcinoma who can be adequately treated by surgery alone; at the same time, it may help to identify a group of patients with laryngeal carcinoma who must be treated with combined modality therapy.

Long-term smoking and alcohol abuse is strongly associated with clonal growths in the upper aerodigestive tract [39]. The data show that the average duration of preoperative smoking was longer in the patients with LOH than in those without LOH; however, the relationship between smoking and LOH will require further study. We speculate that some components of tobacco smoke continue to stimulate the body and promote the occurrence of LOH, and it has been demonstrated that a TP53 mutation is associated with a history of tobacco use [40].

Goodwin observed that the weighted average of 5-year survival in the meta-analysis was 39% in 1,080 patients from 28 different institutions [41]. Using directly calculating method and Kaplan-Meier's method, the 5-year survival rates were 32.14%(9/28)in patients with LOH versus 44.4%(5/9) in patients without LOH in our study. A significant difference between patients with LOH and patients without LOH. The data show that the 5-year survival rates of laryngeal carcinoma are closely related to chromosomal aberrations (LOH), it implies the prognosis of laryngeal carcinoma may relate to chromosomal aberrations.

Unfortunately, we have no prospective data on the postoperative response of tumours with LOH to chemotherapy and radiation therapy. Again, our results confirmed that it would be clinically useful to determine therapeutic strategies for postoperative laryngeal carcinoma.

## Supporting Information

Material S1 **DNA amplification condition for D3S1234, D5S592, D9s171, D9s104, D9S162, D3S1300, D8s261, D8S552, TP53 and D13S317.**
(DOC)
