# Supplementary material for: LOH Detected by Microsatellite Markers Reveals the Clonal Origin of Recurrent Laryngeal Squamous Cell Carcinoma
Source: PLoS One. 2014 Nov 3;9(11):e111857. doi: 10.1371/journal.pone.0111857 (PMC4218824; doi:10.1371/journal.pone.0111857)
Supplement: Material S1 — DNA amplification condition for D3S1234, D5S592, D9s171, D9s104, D9S162, D3S1300, D8s261, D8S552, TP53 and D13S317. (DOC) [file pone.0111857.s001.doc]

DNA amplification condition

D8S261 Cycle parameters for

94℃ 1min 30s, 58℃ 1min 30s, 72℃ 1min 30s, 3 times cycle

94℃ 1min 30s, 56℃ 1min 30s, 72℃ 1min 30s, 5 times cycle

94℃ 1min 30s, 54℃ 1min 30s, 72℃ 1min 30s, 22 times cycle

D9S104 Cycle parameters for

94℃ 1min 30s, 56℃ 1min 30s, 72℃ 1min 30s, 3 times cycle

94℃ 1min 30s, 54℃ 1min 30s, 72℃ 1min 30s, 5 times cycle

94℃ 1min 30s, 52℃ 1min 30s, 72℃ 1min 30s, 22 times cycle

D9s171 Cycle parameters for

94℃ 1min 30s, 56℃ 1min 30s, 72℃ 1min 30s, 3 times cycle

94℃ 1min 30s, 54℃ 1min 30s, 72℃ 1min 30s, 5 times cycle

94℃ 1min 30s, 52℃ 1min 30s, 72℃ 1min 30s, 22 times cycle

D13S317 Cycle parameters for

94℃ 1min 30s, 59℃ 1min 30s, 72℃ 1min 30s, 3 times cycle

94℃ 1min 30s, 57℃ 1min 30s, 72℃ 1min 30s, 5 times cycle

94℃ 1min 30s, 55℃ 1min 30s, 72℃ 1min 30s, 22 times cycle

D8S852 Cycle parameters for

94℃ 1min 30s, 50℃ 1min 30s, 72℃ 1min 30s, 3 times cycle

94℃ 1min 30s, 48℃ 1min 30s, 72℃ 1min 30s, 5 times cycle

94℃ 1min 30s, 46℃ 1min 30s, 72℃ 1min 30s, 22 times cycle

D9s162 Cycle parameters for

94℃ 1min 30s, 56℃ 1min 30s, 72℃ 1min 30s, 3 times cycle

94℃ 1min 30s, 54℃ 1min 30s, 72℃ 1min 30s, 5 times cycle

94℃ 1min 30s, 52℃ 1min 30s, 72℃ 1min 30s, 22 times cycle

D3S1234 Cycle parameters for

94℃ 1min 30s, 59℃ 1min 30s, 72℃ 1min 30s, 3 times cycle

94℃ 1min 30s, 57℃ 1min 30s, 72℃ 1min 30s, 5 times cycle

94℃ 1min 30s, 55℃ 1min 30s, 72℃ 1min 30s, 22 times cycle

D3S1300 Cycle parameters for

94℃ 1min 30s, 59℃ 1min 30s, 72℃ 1min 30s, 3 times cycle

94℃ 1min 30s, 57℃ 1min 30s, 72℃ 1min 30s, 5 times cycle

94℃ 1min 30s, 55℃ 1min 30s, 72℃ 1min 30s, 22 times cycle

D5s592 Cycle parameters for

94℃ 1min 30s, 48℃ 1min 30s, 72℃ 1min 30s, 3 times cycle

94℃ 1min 30s, 46℃ 1min 30s, 72℃ 1min 30s, 5 times cycle

94℃ 1min 30s, 44℃ 1min 30s, 72℃ 1min 30s, 22 times cycle

TP53 Cycle parameters for

94℃ 1min 30s, 50℃ 1min 30s, 72℃ 1min 30s, 3 times cycle

94℃ 1min 30s, 48℃ 1min 30s, 72℃ 1min 30s, 5 times cycle

94℃ 1min 30s, 46℃ 1min 30s, 72℃ 1min 30s, 22 times cycle
